# Selective Cooling of the Brain in Newborn Piglets and Rabbits Using a Novel Nasopharyngeal Method

**DOI:** 10.1186/2197-425X-3-S1-A484

**Published:** 2015-10-01

**Authors:** M Fazel Bakhsheshi, L Morrison, L Keenliside, TY Lee

**Affiliations:** Robarts Research Institute, London, Canada; Lawson Health Research Institute, London, Canada; Western University, London, Canada

## Introduction

Mild hypothermia has become an effective neuroprotective strategy following head trauma, cardiac arrest and neonatal asphyxia. However, cooling the whole body below 33-34°C can induce severe complications; therefore, selective brain cooling (SBC) could minimize adverse effects by maintaining core body temperature at normal values over an extended period of time. Recently, we developed a novel method of SBC and demonstrated its safety and efficacy in a piglet model. the method was based on spraying room temperature or cold air into the nostrils at different flow rates. Pigs possess a carotid rete (a set of small parallel arteries) which is surrounded by the cavernous sinus; together these serve as an effective heat exchanger for the brain. However, in mammals in which the carotid rete is missing such as rabbits and humans, some suggest that there is no effective heat exchange in the cavernous sinus and, consequently, SBC is not efficient in these species.

OBJECTIVE

To evaluate the effectiveness of this approach on rabbits and compare it with previous finding on newborn piglets.

## Methods

Experiments were conducted on six rabbits. Anesthesia were induced and maintained during experiments by ventilation with isoflurane. Body temperature was measured continuously using an esophageal and a rectal temperature probe while brain temperature was measured with an implanted thermometer. Two successive experiments were performed on each animal. in the first experiment, nasopharyngeal brain cooling was initiated by blowing room temperature air from the hospital medical air outlet, at a flow rate of 14-15 L/min into both nostrils for 60 min. the brain was then allowed to gradually rewarm to the baseline temperature. Following rewarming, the second series of measurements and brain cooling was performed in the same manner as the first one but blowing cold air (-7°C) at the same flow rate.

## Results

One hour post cooling with room temperature air at a flow rate of 14-15 L/min, the brain temperature was 34.1 ± 1.2°C which resulted in mean brain cooling rates of 3.7 ± 0.9°C/h. Brain temperature could be reduced more rapidly at mean rates of 5.2 ± 1.9°C/h, while the body temperature as measured by the rectal temperature probe was maintained above 35.5°C during cooling and maintaining period. Mean brain cooling rate was significantly greater with -7°C as compared with room temperature air in both species.

## Conclusions

Results demonstrates clearly that lack of a carotid rete does not reduce the efficacy of SBC with nasopharyngeal cooling. Moreover, the vortex tube cooling system clearly demonstrates ease of use and application, as well as the benefit of portability, important for its adoption to achieve intracranial hypothermia in clinical environment.
